# *Ramomarthamyces octomerus* sp. nov. and Insights into the Evolution and Diversification of *Ramomarthamyces* (Ascomycota, Leotiomycetes, Marthamycetales)

**DOI:** 10.3390/jof10050301

**Published:** 2024-04-23

**Authors:** Jason M. Karakehian, Luis Quijada, Andrew N. Miller, Lothar Krieglsteiner, Hans-Otto Baral

**Affiliations:** 1 Illinois Natural History Survey, University of Illinois at Urbana-Champaign, 1816 South Oak Street, Champaign, IL 61820, USA; 2 Department of Plant Biology, University of Illinois at Urbana-Champaign, 505 South Avenue, Urbana, IL 61801, USA; 3 Departamento de Botánica, Ecología y Fisiología Vegetal, Facultad de Farmacia, Avenida Astrofísico Francisco Sánchez, s/n, Apartado 456, 38200 San Cristóbal de La Laguna, Canary Islands, Spain; 4 Pilzschule Schwäbischer Wald, Brunnenweg 32, 73565 Spraitbach, Germany; 5 Independent Researcher, Blaihofstr. 42, 72074 Tübingen, Germany

**Keywords:** 1 new taxon, amyloid reaction, asci, desiccation-tolerant discomycetes, Garajonay National park, Marthamycetaceae

## Abstract

The apothecial fungus *Ramomarthamyces octomerus* sp. nov. is described from specimens collected in Mediterranean climate regions in southern Portugal, Spain (Canary Islands), and the Dalmatian region of Croatia. Presumably saprobic, *R. octomerus* occurs on intact, decorticated wood of *Laurus novocanariensis* and *Olea europaea*. Ascospores are cylindric-ellipsoid and seven-septate. Surprisingly, in our four-locus phylogenetic analysis (nuSSU, ITS1-5.8S-ITS2, LSU, mtSSU), this fungus clusters among species of *Cyclaneusma*, *Marthamyces*, *Naemacyclus*, and *Ramomarthamyces* in a core Marthamycetaceae clade that circumscribes primarily leaf-inhabiting, filiform-spored species. In addition, the asci of *R. octomerus* possess an amyloid pore, but the reaction varies between specimens collected in the Canary Islands and those collected in Portugal and Croatia. The occurrence of an amyloid reaction in the asci of *R. octomerus* challenges the characterization of Marthamycetales taxa as possessing inamyloid asci. In our discussion we provide background and analysis of these notable observations.

## 1. Introduction

Marthamycetales is a monotypic order with only one family, Marthamycetaceae, which circumscribes species of small, saprobic, or parasitic, apothecial fungi. The order currently circumscribes *Cyclaneusma*, *Marthamyces*, *Mellitiosporiella*, *Mellitiosporium*, *Naemacyclus*, *Phragmiticola*, *Propolina*, *Propolis*, and *Ramomarthamyces* [1,2]. Baral et al. [1] noted that these have been colloquially termed the *Propolis*-like, or “propoloid” fungi. Apothecia develop as lens-shaped primordia within plant tissues and become erumpent at maturity. This action leaves ragged, projecting flaps consisting of degraded plant tissue and hyphae around the exposed hymenium [3], the surface of which is more or less covered in a farinaceous layer that may be white or variously colored. Apothecia are generally long-lived and desiccation-tolerant. There is no excipulum; the subhymenium is seated directly on degraded plant cells. Paraphyses are filamentous with apices that are contorted, or that dichotomously or repeatedly branch. Asci are inoperculate, with apices that are somewhat differentiated or not, dome-like, or truncated-rostrate. Except for *Phragmiticola*, asci of Marthamycetales taxa have been characterized as not reacting in iodine-based reagents. Ascospores are filiform or cylindric-ellipsoid, smooth, hyaline, and aseptate, transversely septate, or muriform. Pycnidial anamorphic states are described for a few taxa, but it is unclear whether these act as conidia or spermatia [1,4,5,6,7,8,9,10,11,12,13,14,15,16,17,18,19,20]. These developmental and morphological features are thought to be adaptations to inhabiting suspended substrates such as bark, dead branches, and dead leaves that may be frequently desiccated and exposed to UV radiation and strong winds [21,22].

Although taxon sampling has been somewhat limited in phylogenetic studies of Marthamycetales, it has been adequate to inform hypotheses regarding the classification, morphology, ecology, and diversity of these fungi. Early phylogenetic analyses of select genera in Leotiomycetes conducted by Lantz and Hustad & Miller [23,24] included *Cyclaneusma*, *Marthamyces*, *Naemacyclus*, and *Propolis*. These genera formed a clade that Baral et al. [1] named Marthamycetaceae. We note that the inclusion of the isolate of “*Mellitiosporium versicolor*” in Lantz and Hustad & Miller [23,24] (and later in Johnston & Park [20]) as representative of that genus is an error arising in Lantz [23] from a misidentified specimen (Lantz 357, UPS F-556815) that the sequence, GenBank no. HM140560, was obtained from. We examined this specimen in the present study and it is *Propolis viridis*. The name of GenBank no. HM140560 has since been corrected to *P. viridis*. Thus, *Mellitiosporium* remains unsequenced. Regardless, the observation given in Baral et al. [1] that both studies had estimated two lineages within Marthamycetaceae that could each be morphologically diagnosed by ascospore shape remained valid. One of these lineages consisted of *Cyclaneusma* + *Marthamyces* + *Naemacyclus* that produce filiform ascospores, and the other lineage consisted of *Propolis* that produces cylindric-ellipsoid ascospores. Recently, Johnston & Park [20] created *Ramomarthamyces* to accommodate a phylogenetic lineage of species formerly placed in *Marthamyces*, but with distinctly branching, rather than propoloid, paraphyses apices. *Ramomarthamyces* clusters in the *Cyclaneusma* + *Marthamyces* + *Naemacyclus* clade. In addition to the morphological diagnosis given in Baral et al. [1] there is an ecological one: *Cyclaneusma*, *Marthamyces*, *Naemacyclus*, and *Ramomarthamyces* circumscribe primarily dead leaf-inhabiting species (*N. fimbriatus* also occurs on female pinecones, and is more frequently collected from this substrate), whereas *Propolis* species occur on dead woody tissues such as wood, bark, and female pinecones ([25], p. 372; [26]). Baral et al. [1] hypothesized that the unsequenced *Mellitiosporiella*, *Phragmiticola*, and *Propolina* might form a clade with *Propolis* based on the similarity of producing cylindric-ellipsoid ascospores, and we note that these genera also fruit on wood. We add *Mellitiosporium* to this list, but we exclude *Phragmiticola* from Marthamycetales based on our preliminary research, which will be presented in a separate publication.

To summarize and form a hypothesis (sequenced genera in bold type): the filiform-spored, dead leaf-inhabiting genera ***Cyclaneusma***, ***Marthamyces***, ***Naemacyclus***, and ***Ramomarthamyces*** form one phylogenetic lineage in Marthamycetaceae, whereas the cylindric-ellipsoid-spored, dead woody tissue-inhabiting genera *Mellitiosporiella*, *Mellitiosporium*, *Propolina*, and ***Propolis*** form another lineage. However, we must revisit this hypothesis in light of a heretofore undescribed fungus found in Europe and Macaronesia that has cylindric ascospores and occurs on wood, but which is phylogenetically placed in *Ramomarthamyces*.

In this contribution, we describe this fungus as *Ramomarthamyces octomerus* sp. nov. based on morphology and multi-gene-based phylogenetic approaches, designating a specimen from La Gomera island, Canary Islands, Spain, as holotype. We also provide a discussion of the unique morphological characters and surprising evolutionary relationships of *R. octomerus* in Marthamycetales.

## 2. Materials and Methods

### 2.1. Specimens, Cultures, and Sequences Used in This Study

Field-collected specimens were air-dried and maintained in cool, dry conditions in the laboratory for study. Specimens used in this study in addition to those of *Ramomarthamyces octomerus* are given below. JMK purchased culture isolates for DNA sequencing from CBS-KNAW (Westerdijk Fungal Biodiversity Institute, Utrecht, The Netherlands). These are listed below.

### 2.2. Additional Specimens Examined

***Cyclaneusma niveum*** GERMANY: Baden-Württemberg, Ulm, in urban park surrounding Wilhelmsburg fortress (Fortress of Ulm), ~223 m SW from SW corner of the fortress; 48.4093303, 9.9802694, 528 m elev. In stand of *Pinus sylvestris*, on fallen leaves of *P. sylvestris*. 21 December 2020. *Ansgar Nartschick 20201221* (coll. no. assigned by JMK). (ILLS 00122400, CBS 149209). Note: teleomorph studied. ***Naemacyclus fimbriatus*** UNITED STATES OF AMERICA: Maine, Washington county, Steuben, grounds of Eagle Hill Institute at 59 Eagle Hill Road, Blueberry Trail near the intersection of the Orchid Trail; 44.456475, −67.928966, 43 m elev. On scales of female cones of *Pinus banksiana*. 25 April 2021. *Joerg-Henner Lotze 21042501* (coll. no. assigned by JMK). (ILLS 00122402, CBS 149212). Note: teleomorph and mitosporic state studied, culture made from teleomorph. Ibid. 22 August 2015. *Jason M. Karakehian 15082202*. (ILLS 00122404, CBS 149214). Note: teleomorph studied. Ibid. 28 May 2017. *Jason M. Karakehian 17052821a*. (ILLS 00122405). Note: teleomorph studied. Rhode Island, Providence county, North Smithfield, Blackstone Gorge, trailside, ~236 m south of parking lot, ~90 m directly east of the Blackstone River; 42.0133, −071.5513, 73 m elev. In small stand of *Pinus rigida*, on scales of fallen, female cones of *P. rigida*. 31 October 2021. *Jason M. Karakehian 21103001*. (ILLS 00122403, CBS 149213). Note: teleomorph studied. Washington county, Narragansett, grounds of the Dunes Club, 137 Boston Neck Road, ~35 m east of parking lot, along walking path on the southern side of the tennis court at the edge of a small woodland; 41.441364, −71.44623, 4 m elev. On scales of female cones of *Pinus resinosa*. 7 June 2013. *Jason M. Karakehian 13060705*. (ILLS 00122406). Note: teleomorph studied. ***Propolis farinosa*** AUSTRIA: Styria, Hartberg, ~4.4 km NNE of Bruck an der Lafnitz, Festenburg, Greith; 47.474, 15.944, 977 m elev. On fallen, decorticated branch of *Fagus sylvatica*. 11 August 2018. *Gernot Friebes GF18081101* (coll. no. assigned by JMK). (ILLS 00122401). Note: teleomorph studied.

### 2.3. Purchased Cultures Sequenced

***Cyclaneusma niveum*** GERMANY: Lower Saxony, Hannoversch Münden, Hedemünden, 51.391898, 9.761136, 141 m elev. On leaves of *Pinus nigra*. 17 June 1971. *H. Peredo*, det. and culture isolation by H. Butin (Butin, 1973). (CBS 495.73). ***Naemacyclus fimbriatus*** SWITZERLAND: *J. Gremmen*. (CBS 289.61). SPAIN: Madrid, near Patones. On scales of female cones of *Pinus pinea*. 25 October 2007. *G. Bills*. (CBS H-20006, CBS 122316).

### 2.4. Morphological Analysis

Photomacrographs of apothecia were made in the laboratory with a Canon EOS 6D digital SLR camera equipped with a Canon EF-S 60 mm or a Canon MP-E 65 mm lens with an attachable ring light. Macromorphological observations of apothecia were made using an Olympus SZX9 or a Motic SMZ-168 stereomicroscope. Apothecia were rehydrated using tap water. Photomicrographs were made using transmitted light microscopy with an Olympus BX51 compound light microscope with 40×, 100×/1.30 oil immersion plan-achromatic objectives, together with an Olympus XC50 5.0-megapixel digital camera and Olympus cellSens Standard 1.14 image processing software. Images were processed in Adobe Photoshop version 25.2. Figures were created in Adobe Illustrator version 28.1. To save space in some figure panels or to improve image readability, some figure panels are montages or are composed of several photomicrographs merged together using Adobe Photoshop’s photomerge function. These figure panels are noted in their respective figure captions. Microscopic drawings were made freehand using a Zeiss Standard 14 microscope with 100×/1.25 oil immersion phase contrast achromatic objective and 15× Euromex wide field oculars.

Mounting media, stains, and reagents for transmitted light microscopy, instructions on identifying mature ascospores in fresh and fungarium material, and definitions of terms such as “euamyloid” and “hemiamyloid” used to describe ascus apex reactions in iodine reagents, follow [27]. Briefly, specimens for crush-mounts or sectioning were hydrated by spraying the dry sample with tap water and keeping it in a moist chamber. SDS Congo red (Cr) (1 g Cr dissolved in 5% sodium dodecyl sulphate in d.i. water) was used to stain cell walls, while phloxine and IKI (ca. 1% iodine [I2] and 3% potassium iodide [KI]) were used to stain cell contents. Both Cr and phloxine were diluted in a drop of water before staining to prevent over-staining. Iodine reagents used to test for amyloid reactions included Melzer’s reagent (MLZ) and IKI. Samples were also pretreated in 10% KOH, then placed in a drop of tap water to rinse the KOH, then mounted in IKI, Melzer’s, phloxine, or Cr. The water rinse prevented precipitation of these stains and reagents and the discoloration of iodine to KI. To test for amyloid ascus rings, we sampled portions of the hymenium from several different apothecia in a given specimen, and mounted these either directly in Melzer’s reagent or IKI or pretreated them first with 2–10% solutions of KOH followed by a water rinse, then mounted them in Melzer’s reagent or IKI. To prevent the curling of hand or microtome sections being treated with KOH, these sections were first mounted in water with a coverglass on top, then the KOH was drawn under the coverglass. If other stains were to be applied, tap water was drawn under the coverglass to rinse out the KOH, then the stain was applied.

To study the structure of apothecia and tissue types following Brummelen [28] (pp. 32–34), longitudinal sections through the midpoint of an apothecium were made by hand-sectioning with the aid of a stereomicroscope or by using a freezing stage mounted to a sliding microtome. Hand sections were made to observe living cells and tissues. The freezing microtome apparatus facilitated making sections that were uniform and thin (~15–25 µm). To make these, pieces of substratum supporting an apothecium were hydrated, soaked in a solution of dilute gum Arabic, and oriented on an electric, water-cooled, freezing stage (Physitemp BFS-5MP) mounted to a sliding microtome. Additional dilute gum Arabic was added to completely envelop the sample in a supportive matrix so it would not be damaged during sectioning. The microtome was set to make ~15 µm sections for routine observation, but 20–25 µm sections or greater were routinely made for species with asci wider than 15 µm. Sections were placed in water on a microscope slide and studied with the aid of a stereomicroscope to select the widest sections, which are from the middle of the apothecium. These were preferred for microscopic study and were manipulated using fine-point paint brushes. The remaining sections were air-dried on the microscope slide, and these were kept with the specimen.

Besides our data from living specimens, we made observations from dead-state asci and ascospores. This allowed us to directly compare our measurements with those given in the taxonomic literature, which are more frequently made from dead-state cells and tissues.

To study mature, living ascospores that were discharged from recently collected apothecia, we followed the procedure outlined in Karakehian et al. [29]. Briefly, recently collected apothecia were hydrated and maintained in a moist chamber with a coverglass suspended over them to collect discharged ascospores. The coverglasses bearing living ascospores were mounted in a drop of tap water on a microscope slide and observed using transmitted light microscopy within a few hours of being discharged.

We used these same microscopy preparations to measure dead ascospores. The ascospores were killed by drawing a drop of KOH or MLZ under the coverglass. Because of this approach, we could not make measurements of individual ascospores in both living and dead states. In other words, any given measurement of a dead ascospore was likely not made from the same, single ascospore in the living state.

### 2.5. Culture Methods

Polysporous cultures were established by means of an ascospore deposit onto Difco potato dextrose agar (PDA) or Difco malt/yeast extract agar (MEYA) following the procedure outlined in Karakehian et al. [29]. Briefly, a recently collected apothecium was hydrated on a piece of wet filter paper on the inner surface of the lid of an inverted Petri dish, allowing discharged ascospores to shoot upward onto the growth media. Three or four apothecia were set up this way in a single Petri dish. Cultures were checked for seven consecutive days under 10× magnification under a compound microscope to monitor ascospore germination and growth, as well as to ensure no contaminants were present. Apothecia used to make cultures were dried and saved in a separate packet that was kept with the specimen. Cultures were archived in long-term storage at 7 °C by placing several 2–3 mm blocks of actively-growing mycelium in 2 mL cryovial tubes filled with sterile distilled water. Cultures were also deposited in CBS-KNAW.

### 2.6. Structures Measured

Apothecium length/width measurements were made from the discs when dried, excluding the marginal flaps (20 discs measured per specimen); values reported are for single, not confluent, apothecia. Stromatic tissue thickness (10 measured per specimen) and periphysoid length (10 measured per specimen) were measured in longitudinal sections at various points above and below the level of the hymenium surface along the entire length of the marginal flaps. Ascospores were measured in both living and dead states (30 measured in each state per specimen). Asci were measured in the dead state; living asci were not available at the time when the specimens were studied. Unless otherwise noted, length/width for all microscopic structures were measured from dead material.

### 2.7. Apothecium Terminology

**Figure 1:**
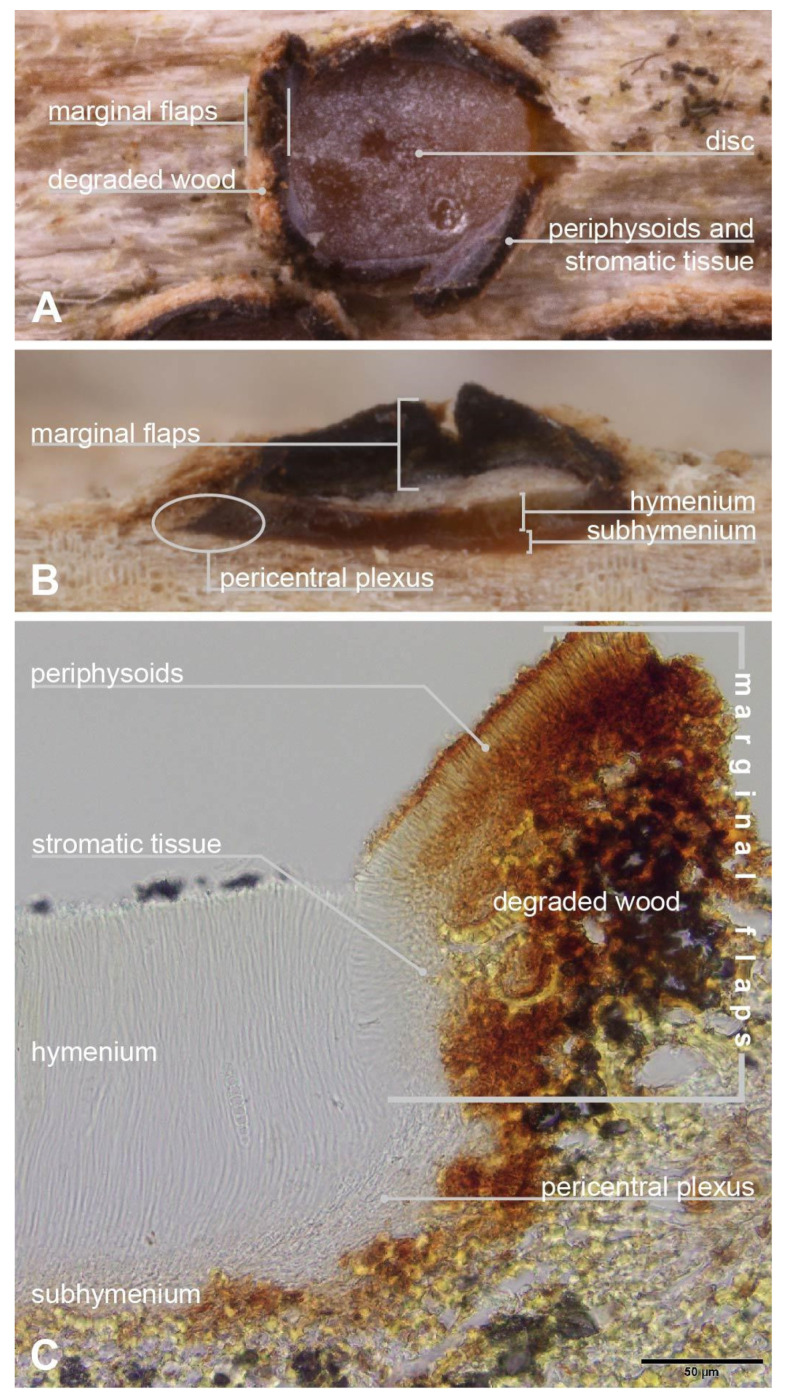
Terminology used to describe the apothecial structure of Marthamycetales species, using apothecia of *Ramomarthamyces octomerus* sp. nov. as an example. (**A**) Photomacrograph of apothecium in face view erumpent through wood substratum (living, hydrated). (**B**) Photomacrograph of a longitudinal section through a mature, open apothecium showing apothecial structure and immersion in wood substratum (living, hydrated; image courtesy of Rubén Negrín Piñero). (**C**) Photomicrograph of a longitudinal section of an apothecium, mounted in Melzer’s reagent. **Specimens studied**: (**A**,**C**) from paratype, ILLS 00122395; (**B**) from holotype, ILLS 00122394.

**Marginal flaps** or **flaps** (Figure 1). The ragged-edged, projecting tissue surrounding the hymenium of an apothecium. Composed of an outer layer of degraded plant tissue infiltrated by loose hyphae that differentiate and give rise to a median layer of stromatic tissue of *textura intricata* that is more or less developed, which in turn gives rise to an inner layer of periphysoids that face the hymenium surface. The periphysoids are arranged like a palisade and are oriented more or less perpendicular to the asci and paraphyses. In a longitudinal section of an apothecium, the bottom of the flaps originate where the periphysoids begin at a point adjacent to the edge of the hymenium and extend upward past the level of the hymenium surface. The periphysoids above the level of the hymenium surface are exposed to the elements and their tips may degrade to some degree, whereas those abutting the edge of the hymenium are more or less protected and intact. The marginal flaps arise because the apothecium develops within the plant tissue, and at maturity the tissue overlying the hymenium splits and hinges back to expose the hymenium. The flaps are somewhat delicate and may remain intact or be broken off at the level of the hymenium surface. We do not consider that variability in the length of the marginal flaps has taxonomic value because they may be broken off and, in round apothecia, because their length is more or less a function of the radius of the apothecium. Presumably, flap thickness might provide an indication of the depth in the substratum that an apothecium developed in, but measurements may be highly variable due to the condition of the outer layer of degraded plant tissue, especially after making longitudinal sections. Moreover, in some cases the thickness of the flaps may be difficult to measure because the plant cells that make up the outer layer might appear to be contiguous with the surrounding substratum. Regardless, we do not consider flap thickness to have taxonomic value and we do not assess it here. Other researchers have used various terms to describe marginal flaps: Fries [4] (pp. 192, 194) and Corda [5] (p. 38) used the term “limbus” (border), Sherwood [30] (pp. 14 and cf. Figure 3c, 15) used “spurious thalline margin” or simply, “thalline margin”, Baral et al. [1] (p. 2) used “lobes”, and Bellemère [3] (pp. 430, 432–433) used “toit” (roof), although this definition excludes the plant tissue. We use “flaps” in this contribution following Johnston [15,16] and Johnston & Park [20] but we note that these authors alternate between “covering layer”, “flaps”, and “margin”. We prefer to use “flaps” because the definition recalls the thinness of the tissue, the hinging action as the tissue tears and peels back as the apothecium opens, and then remains attached only on one side and hanging. 

**Pericentral plexus** or **plexus** (Figure 1B,C) (*peri*: around, *central*: the centrum, *plexus*: an intricate network like nerves or blood vessels). A tissue that radiates outward from the center of the apothecium, below the subhymenium and that, in longitudinal sections made at the midpoint of a mature apothecium, can be observed to differentiate below the edge of the hymenium into a *textura prismatica* that attaches to the lower region of the marginal flaps. Sherwood [30] (p. 10) adapted the term from Letrouit-Galinou [31]. In an analysis of development of discostromatic apothecia, Sherwood [30] (p. 10) explained that the plexus consists of filaments that are attached basally and apically and considered this region to be “meristematic”, that is, the lens-shaped primordial apothecium expands by the lateral extension of this tissue into the surrounding stroma. In apothecia of Marthamycetales, because there is no surrounding stromatic tissue, we consider that the plexus of the primordial apothecium extends into the surrounding substratum of plant cells. Sherwood [30] (p. 10) also considered that the inner face of the plexus is a zone where the paraphyses arise from. We hypothesize that the plexus aids in apothecial development by becoming turgid, along with the hymenial elements at their maturity, to burst open the tissues that overlie the surface of the hymenium; these torn, reflexed tissues become the marginal flaps. In dry apothecia, the plexus cells collapse and this, along with the collapsed hymenial elements, causes the marginal flaps to move inward slightly and cover the surface of the hymenium to a small degree; for the most part, the apothecia of Marthamycetales species never completely close (except in *Cyclaneusma*) as they do in many species of Rhytismatales.

### 2.8. DNA Extraction, PCR Amplification and Sequencing

DNA was extracted using an E.Z.N.A. MicroElute Genomic DNA Kit (Omega bio-tek) following the manufacturer’s instructions. Tissue for DNA extraction was sampled from pure cultures or from apothecia by hydrating the specimen and scraping the hymenium out of several apothecia with a sterile scalpel under a stereo microscope and placing this in an Eppendorf tube filled with extraction buffer and protease from the kit. Eppendorf tubes containing tissue from cultures were lysed at 55 °C in a heating block for 1 hr, whereas those containing tissue from older specimens and/or from small amounts of hymenium were lysed by placing the Eppendorf tubes in makeshift hybridization tubes in a DNA hybridizer oven and rotated at 55 °C overnight, which allowed the material to be constantly agitated. The following primers were employed for PCR amplification and Sanger sequencing: nuSSU: NS1, NS2, NS3, and NS4 [32]; ITS: ITS1F, ITS2, ITS3, and ITS4 [32,33]; LSU: LR3, LR6 [34], LR0R, LR3R [35] (p. S818), https://sites.duke.edu/vilgalyslab/rdna_primers_for_fungi/; mtSSU: mrSSU1, mrSSU2, mrSSU2R, and mrSSU3R [36]. Each 25 µL PCR reaction consisted of 12.5 µL GoTaq G2 Green Master Mix (Promega), 2.0 µL of each 10 µM primer pair, 7.5 µL molecular biology grade water, and 1 µL DNA template. Optimum annealing temperatures (Ta) for each primer pair were determined using IDT (Coralville, IA, USA) OligoAnalyzer (https://www.idtdna.com/calc/analyzer). The following concentration parameters for the reaction mix listed above were also entered: oligonucleotide: 0.5 µM; Na^+^: 50 mM; Mg^++^: 1.5 mM; dNTP: 0.2 mM. The Ta used was the temperature of the primer with the lower of the two melting temperatures (Tm). PCRs were conducted on a BioRad C2000 thermocycler under the following parameters: initial denaturation at 95 °C for 2 min followed by 35 cycles at 95 °C for 45 s, annealing at (Ta) for 45 s and extension at 72 °C for 45 s, with a final extension step of 72 °C for 10 min. PCR products were visualized via gel electrophoresis on a 1% TBE agarose gel. PCR products were cleaned using a Wizard SV Gel and PCR Clean-Up System (Promega). Double-banded PCR products were run on a gel made of TALE buffer (25× stock solution: 60.5 g Tris Base, 14.25 mL glacial acetic acid, 2.5 mL 0.5M EDTA dissolved in 500 mL d.i. water; working solution: 40 mL stock solution in 960 mL d.i. water) and a block of gel containing the band of interest was excised with a clean razor blade on a UV light table and cleaned using this system after melting in binding buffer at 55° C. Sequencing reactions were performed using a BigDye Terminator v. 3.1 Cycle Sequencing Kit (Applied Biosystems), cleaned using ETOH precipitation, and Sanger sequenced in both directions at the Roy J. Carver Biotechnology Center at the University of Illinois Urbana-Champaign. Primers for sequencing reactions included internal, as well as external primers for each locus. Sequence reads were assembled into consensus sequences using Sequencher ver. 5.4.6. LSU sequences were trimmed to the 3′ end of the LR5 primer sequence [34] after consensus assembly to increase sequence quality. Culture isolates purchased from CBS-KNAW were verified by sequencing the ITS region before generating sequences of additional loci.

### 2.9. Handling Sequence Data

A BLASTn [37] search was used to verify the sequence as originating from the intended organism and to identify closely related sequences for inclusion in the alignments. Distances among ITS sequences were evaluated with MEGA 6 [38] using the settings p-distance, transitions + transversions, pairwise deletion. Sequences were accessioned into GenBank [39] citing the respective collection numbers from which they originated. These GenBank accession numbers are listed in Table 1 along with additional sequences downloaded from GenBank that were combined into the dataset.

### 2.10. Phylogenetic Analyses

We performed the phylogenetic analyses using four different DNA regions (nuSSU, ITS, LSU, mtSSU) from representative species of Marthamycetales. We generated 39 new sequences from 4 genera: *Cyclaneusma*, *Naemacyclus*, *Propolis*, and *Ramomarthamyces*. Each gene region was aligned using the Auto algorithm, which selects an appropriate strategy depending on the gene region [40] with MAFFT v. 7.017 [41]. After that, the Gblocks program v. 0.91b [42] was used to identify and eliminate ambiguously aligned regions, using the following relaxed settings [43]: minimum number of sequences for a conserved or flanking position = 24; maximum number of contiguous non-conserved position = 10; minimum length of a block = 5; and gaps in an alignment column allowed in up to half the number of included sequences. Then the 4 genes were concatenated in Geneious. A fixed parameter-rich model (GTR + G + I) was used in lieu of running a test to select the most suitable evolutionary model [44]. Maximum likelihood (ML) and Bayesian Inference (BI) analyses were performed using Geneious v. 6.1.7. [45]. Bayesian inference analyses followed [46], only varying in the number of starting trees (10 million generations) and the tree sampling (every 1000th generation) for BI analysis. Branch support in ML was inferred from 1000 rounds of bootstrap (BS) replicates. We only considered supported clades for ML with bootstraps values ≥ 75% and with posterior probability (PP) values ≥ 0.95 (strongly supported) for BI. Phylogenetics trees were drawn with Geneious and artwork was prepared in Adobe Illustrator CS5.

## 3. Results

### 3.1. Phylogenetic Analyses

**Figure 2:**
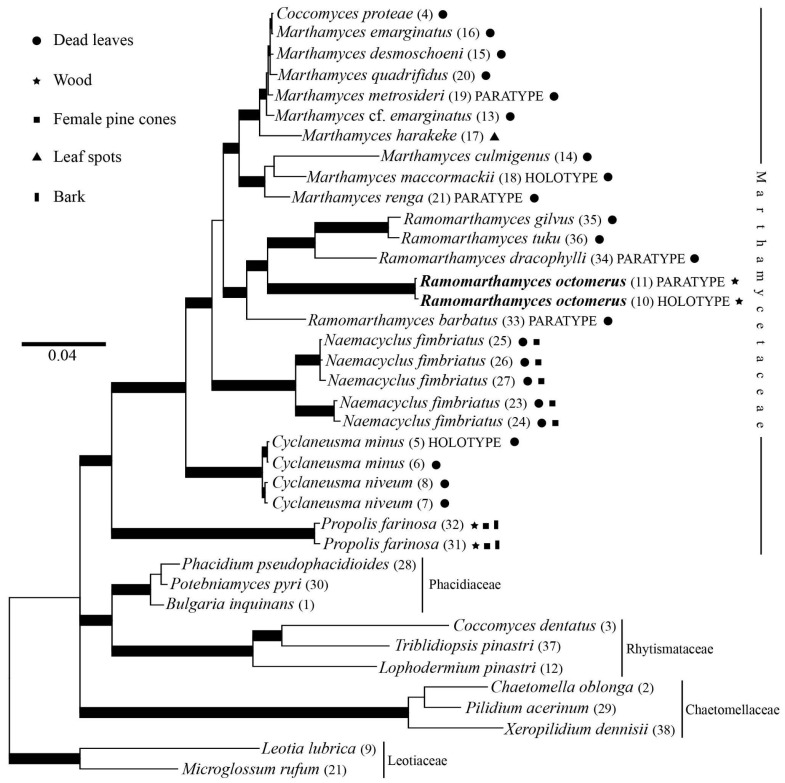
Phylogenetic tree illustrating estimated evolutionary relationships between various taxa of Marthamycetales (not all genera sampled) and *Ramomarthamyces octomerus* (in bold type). Bayesian majority-rule consensus tree based on four-locus concatenated nuSSU, ITS1–5.8S–ITS2, LSU, and mtSSU sequences. Bold branches are well supported (threshold values ≥0.95 for Bayesian, ≥75% for ML). The numbers in the parentheses beside a species name refer to that isolate in Table 1.

The alignment consisted of 3215 characters of which 725 were parsimony-informative, 1035 were variable, and 2180 were constant. The overall topologies of the BI and ML analyses were identical; we only showed the Bayesian consensus tree (Figure 2). The analyses identified different monophyletic, supported clades within the Marthamycetaceae corresponding to *Cyclaneusma*, *Marthamyces*, *Naemacyclus*, *Propolis*, and *Ramomarthamyces*. The two collections of *R. octomerus*, ILLS 00122394 and ILLS 00122395, clustered together in our phylogenetic analyses. The ITS sequences of these were compared in a two-sequence alignment using BLASTn [37] on NCBI (https://www.ncbi.nlm.nih.gov/. They differ by 1 bp (C vs. T) in the ITS1 and by 2 bp (G, C vs. A, T) in the 5.8S, respectively, while no difference occurred in the ITS2. In the LSU D1–D2, 1 bp deviates in the D2 (T vs. C), and in the mtSSU 2 bp deviate in the region near the 5′-end (G, T vs. T, C), respectively. The new, wood-inhabiting species, *R. octomerus*, is nested among the other *Ramomarthamyces* species previously described from leaves. Furthermore, *Ramomarthamyces* is nested among other genera (*Cyclaneusma*, *Marthamyces*, *Naemacyclus*) that are dominated by leaf-saprobic species, although the *Propolis* lineage is saprobic on wood (Figure 2).

### 3.2. Morphological Analyses

We observed that there were differences in the occurrence and quality of the amyloid ascus ring reaction between the specimens of *R. octomerus* collected in the Canary Islands, La Gomera, and those collected in Algarve, southern Portugal, and Korčula island, Croatia. We examined specimens of *R. octomerus* in Melzer’s and IKI, with and without 10% KOH pretreatment. These results are summarized in Table 2. We note that apothecia were mature and living in the holotype from La Gomera (ILLS 00122394) and a paratype from Algarve (ILLS 00122395) because both of these specimens discharged living ascospores onto growth media for culture studies. Of the four specimens mounted directly in Melzer’s reagent, there was an amyloid reaction only in the material from Croatia and partly in the material from Algarve. When mounted directly in IKI, only these two specimens reacted, although the blue reaction ranged from somewhat dingy blue (Figure 5W) to bright blue in the material from Algarve, or dingy reddish gray at high IKI concentration (Figure 7(2E–H) that shows the blue reaction at lower concentration) in the Croatian material. This is in comparison with the bright, clear blue amyloid reactions observed in both of these specimens after pretreatment in KOH (Figure 5S–V [Algarve]), which represents the intermediate type of hemiamyloidity as described by [47]. This is in striking contrast with specimens collected from the laurel forest of La Gomera (ILLS 00122394 and H.B. 6986). Asci from these two collections exhibited a thin apical wall and were negative when mounted directly in IKI (Figures 5Q,R and 7(1E)). Even after KOH pretreatment, the reaction was mostly negative. JMK observed no reaction in any of the mature apothecia sampled from the holotype from La Gomera (ILLS 00122394), only amyloid splotches in the hymenium in a hand section of a senesced apothecium that are presumably the degraded remains of apices of asci that had discharged ascospores and collapsed back among the paraphyses (Figure 6L). LQ observed a weak amyloid apical ring reaction in a very immature ascus with a distinct apical wall thickening (2 µm) and less thickened lateral wall (1–1.2 µm) in the isotype (TFC Mic. 25521), after KOH pretreatment and mounting in Melzer’s reagent (Figure 5X). HOB observed no reaction and no apical wall thickening in H.B. 6986 with IKI even after KOH pretreatment. In conclusion, the presence/absence of an ascus apical wall thickening was consistently correlated with the presence/absence of an amyloid reaction in the specimens studied.

## 4. Taxonomy

***Ramomarthamyces* P.R. Johnst.** In Johnston & Park. 2019. *Mycotaxon* 134: 510–511. 

**Type**: *Ramomarthamyces dracophylli* (P.R. Johnst.) P.R. Johnst. 

**Included species**: *R. barbatus*, *R. dracophylli*, *R. gilvus*, *R. octomerus*, *R. tuku*.

**Emended description**: (based on [15,16,20]). Apothecia in face view 0.3–2 × 0.2–1.2 mm (surrounded by a white, wispy mycelium in *R. barbatus*), round–elongate/elliptic, dehiscence via a stellate splitting of covering layers (or a single, longitudinal slit in *R. tuku*), disc white, pale–bright yellow (grayish-lilac/lavender to medium cool gray or gray-brown in *R. octomerus*), farinaceous layer of the disc absent, thin, or thick, then appearing “felted” or “wooly”; in longitudinal section marginal flaps composed of an outer layer of plant cells interspersed with hyphae, median layer stromatic, composed of densely interwoven hyphae, 4–50 µm thick (well-developed in *R. octomerus*), giving rise to an inner layer of periphysoids 8–15 (12–40 in *R. octomerus*) × 2–5 µm wide; lower wall lacking (or 10–15 µm thick in *R. tuku*); subhymenium 5–20 µm thick; paraphyses 0.8–2 µm wide, more or less dichotomously or many-branched near the apices, apices immersed in the farinaceous substance that covers the hymenium, septation? (septate in *R. octomerus*); asci 115–150 × 7–10 µm (125–190 × 15–20 µm in *R. octomerus*), cylindric (cylindric-clavate in *R. octomerus*), apex truncate-rostrate or broadly rounded, undifferentiated or with slightly thickened wall with a broad, central pore, inamyloid? (amyloidity variable from inamyloid to euamyloid in *R. octomerus*, more often observed after 10% KOH pretreatment), eight-spored; ascospores in all species except *R. octomerus* 75–112 × 1.2–3 µm, filiform, not or slightly tapering toward the poles, bent or coiled upon discharge, 0–1 transverse septa (with a 3.5–4 µm wide swelling immediately above the septum in *R. tuku*), gelatinous caps at poles 2.5–3.5 µm diam, globose or wedge-shaped, in *R. octomerus* 26–42 × 6–10 µm (dead), cylindrical, oblong-obovoid, rarely fusiform, straight or slightly curved, seven transverse septa, multiguttulate, lacking gelatinous equipment. **Additional notes**. All species except *R. octomerus* have an intrahypodermal immersion in leaf tissue, whereas *R. octomerus* is immersed in wood. Apothecia are not associated with bleaching or discoloration of surrounding substratum. 

**Figure 3:**
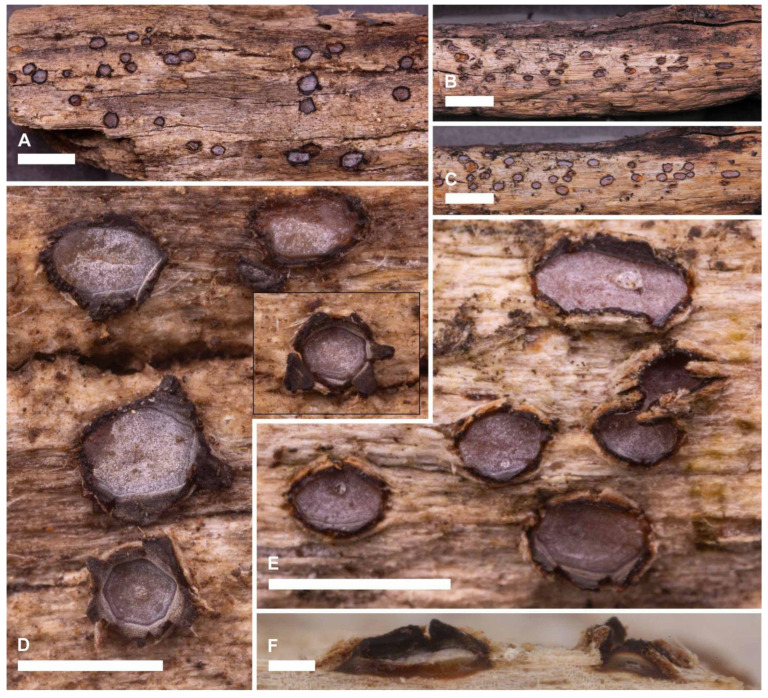
Photomacrographs of apothecia of *Ramomarthamyces octomerus* (all living and hydrated except for B, which is living but dry). (**A**–**E**): Apothecia erumpent through wood substratum, in face view. (**F**): Longitudinal section through two apothecia showing apothecial structure and immersion level in the wood substratum (image courtesy of Rubén Negrín Piñero). **Scale bars**: (**A**–**C**) 5 mm, (**D**,**E**) 2 mm, (**F**) 0.5 mm. **Specimens studied**: (**A**,**D**) (with insert), (**F**) from holotype, ILLS 00122394; (**B**,**C**,**E**) from paratype, ILLS 00122395.

**Figure 4:**
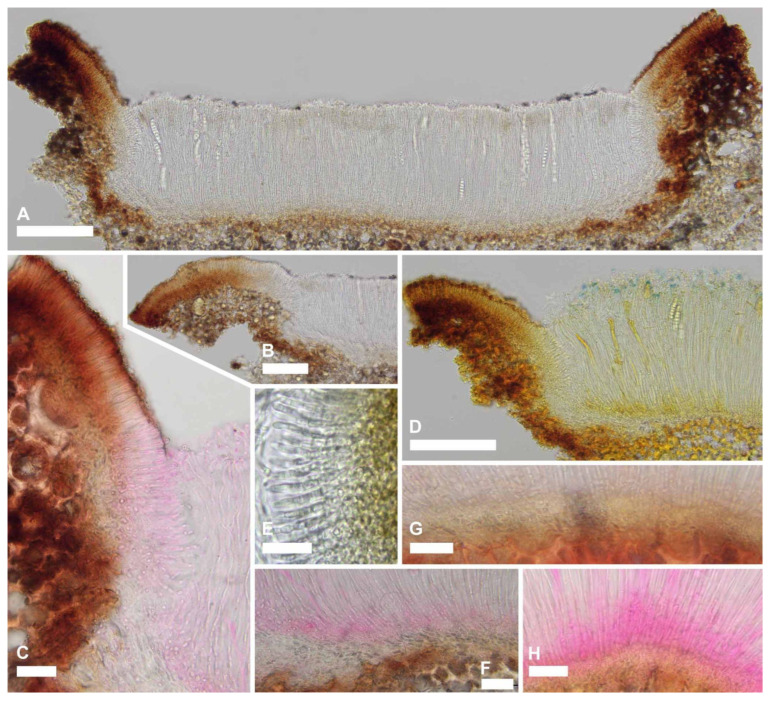
Photomicrographs of longitudinal sections of apothecia of *Ramomarthamyces octomerus* (all from dead material except E; A–C photomerged). (**A**,**B**): Image showing apothecial structure and tissue pigmentation. (**C**): Detail image showing the structure of a marginal flap and pericentral plexus, the uppermost region of which connects to the wood cells at the lowermost region of the flap). (**D**): Detail image showing the uppermost region of the hymenium with amyloid ascus rings, and in the lowermost region of the hymenium showing strong staining of the cell contents of the lowermost cells of the paraphyses, as well as ascogenous hyphae and developing asci (also observed in C,F,H). (**E**): Detail image showing periphysoids (living). (**F**–**H**): Detail images showing (from top to bottom) the lowermost region of the hymenium, the subhymenium, and wood cells, illustrating how the paraphyses and pericentral plexus (shown in panel F) arise from the subhymenium; degraded wood cells directly underlie the subhymenium and plexus. **Reagents and stains used**: (**A**,**B**,**E**) in tap water; (**C**,**F**,**H**) pretreatment in 10% KOH followed by water rinse followed by phloxine; (**D**) pretreatment in 10% KOH followed by water rinse followed by IKI; **G** pretreatment in 10% KOH followed by water rinse followed by Congo red. **Scale bars**: (**A**,**B**,**D**) 100 µm; (**C**,**E**–**H**) 20 µm. **Specimens studied**: (**A**,**C**,**D**,**F**–**H**) from paratype, ILLS 00122395; (**B**,**E**) from holotype, ILLS 00122394.

**Figure 5:**
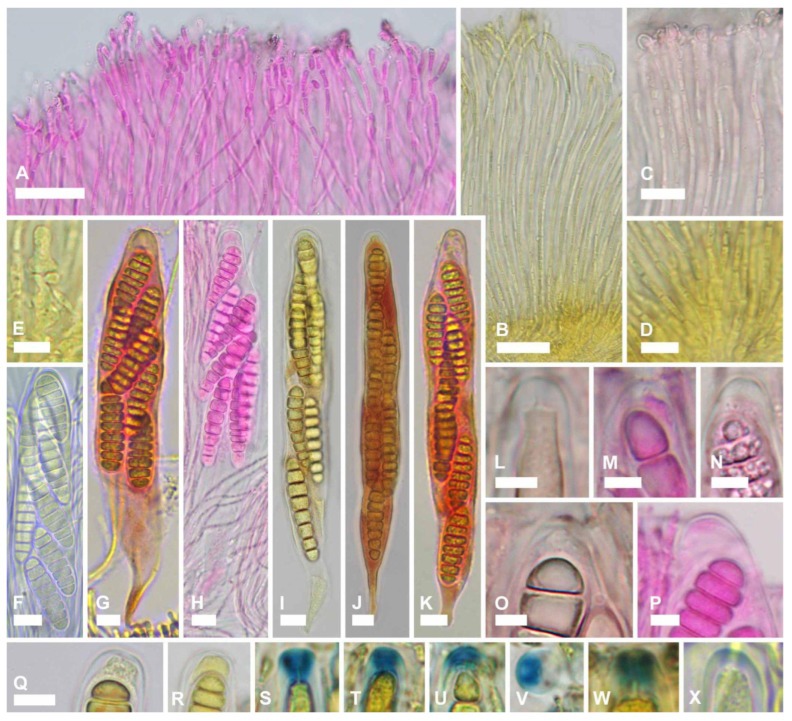
Photomicrographs of paraphyses and asci of *Ramomarthamyces octomerus* (all from dead material). (**A**–**D**): Paraphyses. (**E**–**X**): Asci. (**A**): Upper regions of paraphyses, showing septation, branching, and apices. (**B**): Full-length image of paraphyses arising from the subhymenium, showing septation, branching, and apices. (**C**): Paraphyses agglutinated in gel showing anastomosis and knob-like apices. (**D**): Paraphyses branching near the bases. (**E**): Crozier. (**F**–**K**) Mature asci (note thin ascus walls in the lower half of the asci in G,H). (**L**–**P**): Ascus apices in progressive stages of ascus development with L being the least mature ascus (no ascospore delimitation, thickened apex), (**M**) ascus becoming mature (ascospores forming septa, apex thinning), (**N**–**P**) mature asci (ascospores septate, apices thinning). (**Q**–**X**): ascus apices mounted in iodine reagents, showing amyloid apical ring in **S**–**X**. **Reagents and stains used**: (**A**,**C**,**H**,**M**,**N**,**P**) pretreatment in 10% KOH followed by water rinse followed by phloxine; (**B**,**D**,**E**,**I**,**J**,**Q**–**V**) pretreatment in 10% KOH followed by water rinse followed by IKI; (**F**,**X**) pretreatment in 10% KOH followed by water rinse followed by Melzer’s; (**G**,**K**,**L**,**O**) pretreatment in 10% KOH followed by water rinse followed by Congo red; (**W**) IKI. **Scale bars**: (**A**–**D**) 20 µm; (**E**,**L**–**X**) 5 µm; (**F**–**K**) 10 µm. **Specimens studied**: (**A**,**B**,**D**,**E**,**G**–**K**,**P**–**R**) from holotype, ILLS 00122394; (**C**,**L**–**O**,**S**–**W**) from paratype, ILLS 00122395; (**F**) from paratype, TFC Mic. 25520, (**X**) from isotype, TFC Mic. 25521.

**Figure 6:**
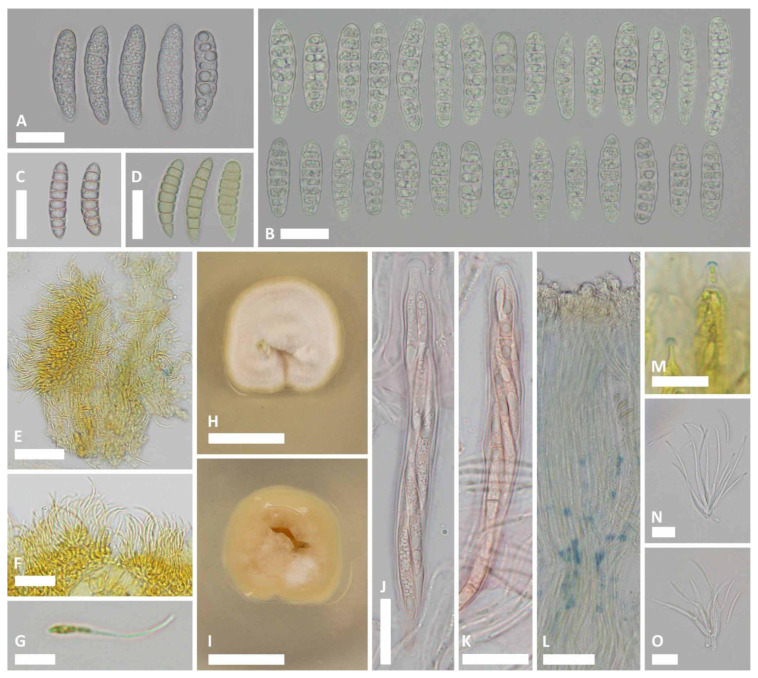
Photomicrographs of ascospores, anamorph(?), pure culture, immature asci, and hymenium of senescent apothecium of *Ramomarthamyces octomerus*, including images of asci and anamorph of *Naemacyclus fimbriatus*. (**A**–**L**): *R. octomerus*. (**M**–**O**): *N. fimbriatus*. (**A**–**D**): Ascospores discharged from living apothecia (photomontages). (**A**,**B**): Living, mature ascospores (note lipid pattern [multiguttulate] and turgid cells that produce constrictions at the septa). (**C**,**D**): Dead, mature ascospores (note obscured lipid pattern and loss of turgidity). (**E**–**G**): Associated mitosporic state? (**H**,**I**): Mycelium in pure culture; face-view (**H**) and reverse (**I**). (**J**,**K**): Immature asci showing immature ascospores. (**L**): Detail of longitudinal section of senesced apothecium showing amyloid apical rings of degrading, collapsed asci in the hymenium. (**M**): Amyloid rings of asci of *N. fimbriatus*. (**N**,**O**): *Eriosporopsis albida*, considered to be the anamorph of *N. fimbriatus* (living material). **Reagents and stains used**: (**A**,**B**,**N**,**O**) in tap water; (**C**,**J**,**K**) pretreatment in 10% KOH followed by water rinse followed by Congo red; (**D**–**G**): in Melzer’s; (**L**,**M**): pretreatment in 10% KOH followed by water rinse followed by IKI. **Scale bars**: (**A**–**E**,**J**–**L**) 20 µm; (**F**,**M**–**O**) 10 µm; (**G**) 5 µm; (**H**,**I**) 1 cm. **Specimens studied**: (**A**,**C**–**K**) from paratype, ILLS 00122395; (**B**,**L**) from holotype, ILLS 00122394; (**M**–**O**) from ILLS 00122402.

**Figure 7:**
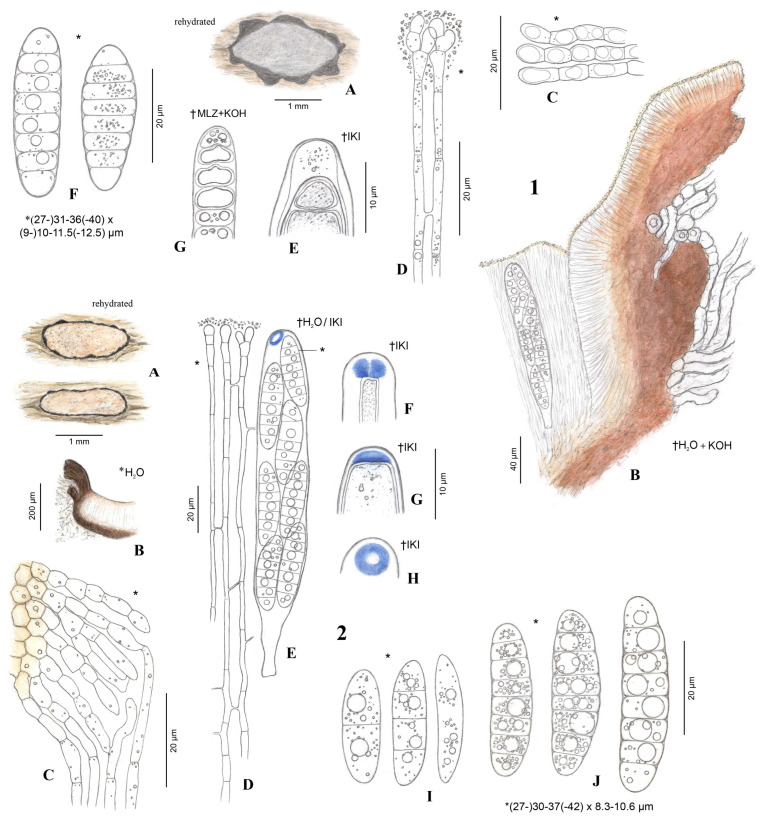
Drawings of apothecia and microscopic features of *R. octomerus* made by HOB. Symbols used: *: living state (in H_2_O), †: dead state. (**1**): H.B. 6986 (La Gomera), (**2**): H.B. 6690 (Croatia). (**1A**,**2A**): Apothecia in face view (hydrated). (**1B**,**2B**): Median sections of apothecia. (**1C**,**2C**): Periphysoids in middle (**1C**) and lower (**2C**) part of marginal flaps (containing vacuoles and minute LBs), in 2C showing connection to lateral paraphyses. (**1D**,**2D**): Paraphyses, with anastomoses and granular exudate around apex. (**2E**): Mature ascus with eccentric apical ring. (**1E**,**2F**–**H**): Ascus apices in dead state (stained with IKI); (**1E**): mature inamyloid ascus with subapical wall thickening and apically thin wall; (**2F**–**H**): asci with amyloid apical ring; (**2F**): immature ascus with laterally thickened wall; (**2G**–**H**): submature asci with thin lateral wall (**2H** seen from top). (**1F**,**2J**): Mature living ascospores, containing small to large LBs, septa with small lateral thickenings. (**2I**): Immature living ascospores, septa not laterally thickened. (**1G**): Mature dead ascospore, lipid content distorted (confluent), septa with stronger lateral wall thickenings.

***Ramomarthamyces octomerus*** Karakehian, Quijada, L.G. Krieglsteiner, Baral sp. nov. Figure 1, Figure 3, Figure 4, Figure 5, Figure 6 and Figure 7.

MycoBank no.: MB853145

**Etymology**: “*octomerus*” refers to the mature ascospores that are divided by cross septa into 8 cells.

**Typification**: SPAIN: Canary Islands, La Gomera, Parque Nacional de Garajonay, W of Mirador Risquillos de Corgo, Raso de la Bruma; 28.1473, −17.2895, 1090 m elev., Laurel forest, on decorticated wood of *Laurus novocanariensis*. 2023. *R. Negrín Piñero RN-23031801*. (Holotype ILLS 00122394; isotype TFC Mic. 25521; ex-holotype culture CBS 150560). GenBank: PP576071 (nuSSU), PP576090 (ITS), PP576080 (LSU), PP554494 (mtSSU). 

**Other specimens examined**: CROATIA: Korčula, Badija island, NE of Turističkosportski center, below Crkva sv.; 42.9535, 17.1652, 45 ± 5 m elev. In south-exposed semi-shaded macchia on calcareous soil, on decorticated, bleached branch stub of *Olea europaea* attached to living tree. 8 June 2000. *HOB H.B. 6690*. PORTUGAL: Algarve (Faro), near Amorosa (village), west of São Bartolomeu de Messines; 37.2499, −8.325564, 200 m elev. In semi-cultured dry shrub- and heathland on calcareous soil, on fallen, decorticated branch of *Olea europaea*. 22 February 2023. *L. & K. Krieglsteiner ALGARVE-1100*. (ILLS 00122395; culture CBS 150559). SPAIN: Canary Islands, La Gomera, Parque Nacional de Garajonay, SW of Chorros de Epina; 28.15944, −17.30195, 1000 m. In north-exposed laurel forest (Fayal-brezal) with *Laurus novocanariensis* and *Ilex canariensis*, on decorticated branch of *Laurus novocanariensis* (as *L. azorica*). 28 April 2001. *E. Beltrán-Tejera* [vid. R. Galán & HOB]. (TFC Mic. 10065, H.B. 6986, AH 59941). Ibid., NNW of Los Baranquillos; 28.1545, −17.3060, 1000 m. In Fayal-brezal with *Laurus* and *Ilex canariensis*, on decorticated wood of *Laurus novocanariensis*. 29 October 2023. *R. Negrín Piñero RN-23102901* (TFC Mic. 25520). Ibid, W of Mirador Risquillos de Corgo, Raso de la Bruma; 28.1475, −17.2897, 1085 m elev. On decorticated wood of *Laurus novocanariensis*. 15 April 2014. *R. Negrín Piñero RN-14041504*. ibid; 28.1472, −17.2902, 1070 m elev. On decorticated wood of *Laurus novocanariensis*. 20 January 2024. *R. Negrín Piñero RN-24012001*. Ibid, SW of Montaña de Tobares, Agua de Los Llanos; (approximately) 28.1397, −17.2389, 1000 m elev. In *Lauro novocanariensis-Perseetum* indicae with *Picconia excelsa*, on decorticated branch of *L. novocanariensis* (as *L. azorica*). 28 April 2001. *E. Beltrán-Tejera* (TFC Mic. 10068, AH 59940).

**Description**: **APOTHECIA IN FACE VIEW** (Figure 3 and Figure 7): **size** 0.6–2.0(−2.8) × 0.4–1.2(−1.5) mm, size not changing considerably when hydrated, remaining open when dry; **shape** elongate, occasionally round, ends blunt, more or less following the orientation of the wood grain—straight when this is straight or slightly curved when this is twisted; **habit** erumpent; **fruiting pattern** gregarious to scattered, solitary, rarely confluent; **disc** when dry or hydrated grayish-lilac/lavender to medium cool gray or gray-brown, the hymenial surface covered by a thin, white pruina that may be easily scraped away when hydrated to reveal the orange to dingy amber colored hymenium beneath, plane when dry or plane to slightly convex when hydrated, in older apothecia with short splits when dry; **marginal flaps** encircling the hymenium, projecting well above the hymenium surface, reflexed, with lacerate edges, appearing more or less thick and two-layered with an outer layer of pale woody tissue and an inner, dark-brown–black layer of fungal tissue that is a leathery, matte texture, with the white pruina on the hymenial surface more or less extending up the inner surface of the flaps and gradually thinning toward the outermost edges; **apothecium texture** fleshy, no parts carbonized; **hymenium texture** waxy-sticky when hydrated, tough when dry. **APOTHECIA IN LONGITUDINAL SECTION** (Figure 4 and Figure 7) (observed in water): **marginal flaps** degraded wood cells near the stromatic tissue having a light- to dark-brown or black inner region that extends down the flanks of the apothecium to below the subhymenium, gradually becoming a pale, narrow band, stromatic tissue distinct, thick, devoid of wood cells, composed of a dense *textura intricata* (?*epidermoidea*) that extends from the top edges of the flaps down to just above the level of the subhymenium, above the level of the hymenium surface the hyphae are surrounded by extracellular pigment so that the tissue appears light- to dark-brown or even black near the top edges of the flaps, below the level of the hymenium surface hyphae not surrounded by extracellular pigment, 6–47(−52) µm thick, giving rise to periphysoids that are septate, occasionally branching, when living thin-walled and slightly to strongly constricted at septa, 22–40 × 2.5–4 µm, when dead thick-walled and constrictions lost, 17–36 × 1.8–3 µm, above the level of the hymenium surface in a tight, palisade-like organization, more or less agglutinated in gel, tips somewhat degraded, hyphae surrounded by extracellular pigment so that this tissue appears light- to dark-brown, darkening more near the top edges of the flaps, below the level of the hymenium surface their palisade-like organization is looser and fans out slightly, not agglutinated in gel, tips intact, terminal cells when living 4.5–12 × 2.5–4 µm, when dead 2–3 µm wide, hyphae not surrounded by extracellular pigment, lowermost periphysoids connected with their tips to the tips of lateral paraphyses; **pericentral plexus** arising from below the subhymenium and fanning out into a distinct tissue of *textura prismatica* that attaches to the degraded wood cells and stromatic tissue at the bottom of the marginal flaps, lacking extracellular pigments, somewhat resistant to cytoplasmic stains; **subhymenium** 5–15 μm thick, hyaline or pale brown or bright ochre, somewhat resistant to cytoplasmic stains, composed of dense more or less square–rectangular cells that give rise to paraphyses, resting directly on a layer of degraded wood cells and arising from the loose, pigmented hyphae that intersperse among these, contiguous with the tissue that forms the plexus, with ascogenous hyphae in the uppermost region; **hymenium** 158–194 μm thick, hyaline, surface covered with a thin layer of pruina, no reaction in any vegetative structures in Melzer’s or IKI even after 10% KOH pretreatment and water rinse. **HYMENIAL ELEMENTS** (Figure 5, Figure 6 and Figure 7): **paraphyses** 1–2 µm wide along their main part, filamentous, septate along their length but more densely so near the base and tip, cells in middle part (living) 13–35 × 1–2 µm, more or less surrounded by gel along their entire length, occasionally branching at the base, anastomoses frequent and occurring anywhere along their length, dichotomously branching near the apices or not, apices simple, or terminating in a slight to moderate, capitate–clavate enlargement, upper 2–3 terminal cells when living 3.5–7.5 × 2.5–4.5 µm, when dead 1.3–2.4 µm wide, apices encrusted in a hyaline granular exudate (pruina); **asci** (all observations from dead asci) 93–177 × 12–25 µm, arising from croziers, cylindrical to cylindric-clavate, typically eight-spored although there may be fewer by abortion, ascospores biseriately arranged, rarely uniseriate (then ~12 µm wide), lateral walls of dead immature asci of varying thickness, becoming thin with maturity and then nearly transparent below the midpoint of the ascus, apices inamyloid or euamyloid to slightly hemiamyloid (blue but turning reddish-gray at high iodine concentration) depending on the collection, specimens with inamyloid asci with thickened subapical wall and thin apical wall (0.2–0.5 µm), specimens with amyloid asci when immature with 3.3–4.5 µm thick apical wall, partly pierced by a narrow extension of the cytoplasm (apical chamber) that is surrounded by an amyloid ring (may require pretreatment in KOH), when mature 1.8–2.6 µm thick apical wall, the apical chamber becoming a broad, central pore, apical ring terminal or slightly to sometimes highly eccentric, mode of dehiscence via the apical ring or via a rupture of the thin inamyloid apical wall; **ascospores** living: 27–52 × 7.4–12.5 µm, dead: 23.5–44.5 × 7.5–13 µm, predominantly cylindrical, sometimes or often with the lower half tapering slightly toward the base, occasionally oblong-obovoid, rarely fusiform, straight or slightly curved, typically eight-celled (seven transverse septa) but fewer or more cells may sometimes be observed, poles obtuse or sometimes subacute or with a very short, acute projection, smooth, hyaline, lacking gelatinous structures, not reacting in KOH or iodine based reagents even after KOH pretreatment, thin-walled, living cells typically multiguttulate or with one large guttule and a few smaller ones, constricted at the septa (the deepest constriction often at the middle, primary, septum), when dead constrictions are lost, septa thin-walled but with a small triangle-shaped lateral thickening at the constriction that may be slightly thicker in dead spores, ascospore development microcephalic (non-graphidean). **CULTURE CHARACTERISTICS** (Figure 6H,I): On PDA (polysporous) colony in face-view slightly humped in the center, tomentose, white, margin entire, in reverse-view cream colored, texture somewhat leathery, growth slow at 1.5–1.7 cm in 12 weeks. Mitospores not observed in culture.

**Additional notes**: **Substratum surface** more or less bleached, no algae, wood relatively hard and intact. **Co-occurring fungi**: *Propolis viridis*, *P. farinosa*, *Berkleasmium conglobatum*, *Hysterium* sp., *Mycosphaerella* sp., *Orbilia vinosa*. **Seasonality** unknown, presumably year-round. **Apothecia** are long-lived and desiccation-tolerant. In older apothecia the pruina covering the hymenial surfaces and the inner surface of the marginal flaps may become worn away, especially in the latter tissue, and the marginal flaps themselves may be broken off to the level of the hymenial surface so that the disc appears to be encircled by a black border. In long-dead apothecia the hymenium and marginal flaps are entirely degraded, leaving a shallow concavity in the wood in the shape and size of the apothecium, the inner surface of which may have an amber or dark brown colored sheen that is presumably the remains of the subhymenium. **Periphysoids** above the level of the hymenium surface have degraded tips so that this tissue is somewhat narrower when observed in longitudinal sections than the periphysoids below the level of the hymenium surface, which have intact tips. **Hymenium**. We observed that in apothecia of the specimen from Algarve, Portugal (ILLS 00122395), the asci and paraphyses were strongly agglutinated in gel that rendered them impossible to separate without destroying them, even after treatment in 10% KOH. **Mitosporic state(?)**. We observed a possible mitosporic state of *R. octomerus* in a crushed hand-section of an apothecium from of the specimen from Algarve, Portugal (ILLS 00122395) (Figure 6E–G). We use the neutral term “mitosporic” because we do not know if the spores function as conidia or spermatia. The sporoma may have been located in the woody substratum below the apothecium and we presume that it was pycnidial, probably with hyaline walls. The mode of mitosporogenesis was blastic-phialidic, with phialides arranged in a palisade-like layer and not branching(?), hyaline, 5–6 × 1 µm. Mitospores filiform, hyaline, smooth, straight, or slightly curved, 10–14 × 0.5–1 µm.

## 5. Discussion

### 5.1. Phylogenetic Placement

Phylogenetic analyses of the specimens from Algarve, Portugal and La Gomera, Canary Islands yielded surprising results. The paratype from Algarve (ILLS 00122395) and the holotype from La Gomera (ILLS 00122394) are phylogenetically very close (deviations: 1 bp and 1 gap in nuSSU V1–V5, 3 bp in ITS, 1 bp in LSU D1–D2, 2 bp in mtSSU). The genetic similarities were unexpected given morphological differences between these collections in the amyloid reaction of the apical ring (cf. Table 2), hymenial elements firmly agglutinated in gel or not, and the habitat of growing on different plant genera in different geographical regions at different altitudes. Although the observed molecular deviations are few (0.6% in ITS), more sequences should be generated to clarify a possible correlation between these molecular deviations and the observed morphological and ecological differences.

Our phylogenetic results also demonstrate that this dead wood-inhabiting, broadly cylindrical-spored fungus is not sister to *Propolis* which is ecologically and morphologically similar, as we had originally hypothesized, but it is more closely related to *Cyclaneusma*, *Marthamyces*, *Naemacyclus*, and *Ramomarthamcyes* which inhabit leaves and produce filiform ascospores. In comparison with the high similarity of our two sequences, ITS similarities using BLASTn [37] between *Ramomarthamyces* and these genera range at around 84.5–86.5% (*Ramomarthamyces* spp.), 84–85% (*Naemacyclus fimbriatus*), 82.5–83.5% (*Marthamyces* spp.), and 82–82.5% (*Cyclaneusma minor* and *Coccomyces proteae*), and LSU D1–D2 similarities at 95.5–96% (*Marthamyces* spp. and *Ramomarthamyces barbatus*, but also *Coccomyces proteae*).

Finally, *Naemacyclus fimbriatus* isolates from Spain and Switzerland formed a well-supported clade that is sister to isolates from northeastern North America. This result suggests that these could represent two geographically distinct species. We are uncertain if the basionym of *N. fimbriatus*, *Stictis fimbriata* Schwein., has been properly typified (in [7]?). Based on the protologue of this name [48], original material would originate from Camden, Kaighn’s Point (as “Kaign’s Point”), NJ, USA. The earliest synonym of *N. fimbriatus* based on European material is *Propolis pinastri* Lacroix, which is described from French material (in [49], species no. 791). We are also uncertain whether this name has been properly typified. An examination of the types of *S. fimbriata* and *P. pinastri*, as well as morphological and phylogenetic analyses of additional North American and European specimens, will be necessary to determine whether European isolates may be referable to *P. pinastri*, and this name ultimately transferred to *Naemacyclus*.

### 5.2. On the Placement of Our New Species

The results of our phylogenetic analyses (Figure 2) raised the question as to whether or not we should create a new genus for the new species described in this contribution. We considered two options that are described and analyzed as follows:

The first option was that we treated the *Ramomarthamyces* clade as paraphyletic, consisting of a “core” *Ramomarthamyces* clade that included the type, *R. dracophylli*, along with *R. gilvus* and *R. tuku*. Here, our new species and *R. barbatus* were separate lineages with our new species being sister to the core clade. We could either create a new genus to accommodate the new species and leave *Ramomarthamyces* as paraphyletic or create this new genus and transfer *R. barbatus* to another new genus. This latter solution would maintain a monophyletic *Ramomarthamyces* that circumscribed the three species in the core clade: the option of creating a new genus only for our new species and leaving *Ramomarthamyces* paraphyletic being undesirable.

To explore this option, we first assessed the morphological differences between our new species and species of *Ramomarthamyces*. Setting aside differences in plant associates, these included the occurrence on decorticated wood rather than dead leaves, larger apothecium size, and ascospores that are cylindric-ellipsoid rather than filiform, with mostly seven septa rather than one, and lacking gelatinous equipment rather than having gelatinous caps at the ascospore poles. However, we were unable to critically assess ascus amyloid reactions among *Ramomarthamyces* species because these are under-studied. Johnston [15] reported an inamyloid ascus apex in *R. dracophylli* in material rehydrated in 3% KOH and mounted in Melzer’s reagent, but we are uncertain whether *R. gilvus*, *R. barbatus*, or *R. tuku* were tested for ascus iodine reactions [16,20]. The protologue of *R. barbatus* is published along with a redescription of *R. gilvus* in Johnston [16], and in the Materials and Methods section of this paper, Johnston describes that specimens were rehydrated in 3% KOH and mounted either in this or lactic acid; no mention of iodine-based reagents is made, and iodine reactions are not given for these two species. This is also the case in Johnston [20] where the protologue of *R. tuku* is published. We checked the protologue of the basionym of *R. gilvus*, *Naemacyclus gilvus* in Rodway [50], and there is no information on ascus iodine reactions there either.

We then assessed whether *R. barbatus* was morphologically distinct from the three species comprising the core *Ramomarthamyces* clade. We studied the protologues and expanded descriptions of *R. barbatus*, *R. dracophylli*, *R. gilvus*, and *R. tuku* given in Johnston [15,16], Johnston & Park [20], and Rodway [50]. Ascus and ascospore size more or less overlapped, but *R. barbatus* appears to be distinctive from the other species in two ways. First, the apothecia are “surrounded by [a] white, wispy mycelium on [the] leaf surface, sometimes [with] several ascomata surrounded by [a] single patch of mycelium, up to 10 mm across” [16]. Furthermore, Johnston [16] noted “Although often well developed, this mycelium can be very sparse, or even absent in some cases”. Indeed, the character was notable enough to warrant the epithet *barbatus*, which is Latin for “bearded”. Second, the marginal flaps (termed as “covering layer”) are composed of a median layer of globose cells 3–4.5 µm diam, whereas in the other species this layer is described as hyphal. We note that Johnston [16] examined several specimens from Tasmania and Victoria, Australia.

The second option was to do nothing and treat the *Ramomarthamyces* clade as monophyletic. Here, we would place our new species in *Ramomarthamyces* and name no new genera. Despite the aforementioned differences in ecology and morphology between the species in this clade, there are morphological similarities that would support this option. These include the erumpent habit, gross appearance of the apothecia with the marginal flaps encircling the hymenium, and the more or less thick pruina covering the apothecial disc, apically branching paraphyses, and ascus apices that when mature are somewhat thick-walled with a broad central pore (this is described in *R. barbatus*, *R. dracophylli*, and *R. gilvus*). We note that considerations surrounding the delimitation of new genera based on differences in ascospore shape and septation patterns should be approached with caution because in some taxa, these characters are unreliable for this purpose [51].

We consider that following option two is the more appropriate approach considering the results of our phylogenetic and morphological analyses. We choose to retain *R. barbatus* in *Ramomarthamyces* because we are uncertain whether the white mycelium surrounding apothecia of *R. barbatus* and the globose, rather than hyphal, cells of the median layer of the covering flaps are significant morphological differences to warrant the creation of a new genus to accommodate this species. Concerning our new species, we are uncertain if the morphological characteristics mentioned above are significant enough to warrant the creation of a new genus in light of the results of our phylogenetic analysis. And, as previously noted, ascospore shape and septation patterns may not be reliable in the delimitation of genera; another *Ramomarthamyces* species, *R. tuku*, possesses an arguably distinctive morphology in ascospores with a small swelling above the median septum, but this species falls within the “core” *Ramomarthamyces* clade sister to *R. gilvus*, which does not share this apomorphy. The as yet unsequenced *Marthamyces dendrobii* possesses an ascospore morphology similar to *R. tuku*. In any case, it is remarkable that the ITS distance between our new species and the species of *Ramomarthamyces* is distinctly higher (p-distance 20–29%) compared to the distance among the species of *Ramomarthamyces* that ranges from 5.5–16%. For now, we consider that maintaining a monophyletic *Ramomarthamyces* is the simpler and more conservative conclusion pending additional taxon and gene sampling, which may ultimately shed additional light on the question.

### 5.3. Notes on Life History

#### 5.3.1. Trophic Mode

Species of *Ramomarthamyces* are presumed to be saprobic [15,16,20]. We presume that *R. octomerus* is saprobic as well because it fruits on dead wood and grows in culture. More broadly in Marthamycetales, most taxa are presumed to be saprobic but notable exceptions include *Cyclaneusma minus*, which is a widespread needle-cast pathogen of *Pinus* species [13], and *Marthamyces culmigenus* and *M. harakeke* that are pathogenic on various genera of Poaceae and on *Phormium tenax*, respectively [17,20].

#### 5.3.2. Endophytic States

In our BLAST searches of an ITS sequence from *R. octomerus*, we did not find matches to sequences derived from endophytes. We have not conducted a search using sequences from other *Ramomarthamyces* species. More broadly in Marthamycetales, Johnston [16] speculated that *Marthamyces* species possessed endophytic states. Recently, these have been demonstrated to occur in *Marthamyces renga* [52]. We observed that several sequences of endophytes from leaves of *Metrosideros excelsa* trees in San Francisco, CA, USA [53] cluster with sequences of *Marthamyces metrosideri* from New Zealand in the Tree View option in BLAST [37]. A full assessment of marthamycetalean endophytes has not been conducted.

#### 5.3.3. Mitosporic States

JMK searched the surface of the substratum in the holotype (ILLS 00122394) and a paratype (ILLS 00122395) specimen of *Ramomarthamyces octomerus* and did not find any sporomata that could be a potential mitosporic state (we use the neutral term “mitosporic” because we do not know if these spores function as conidia or spermatia). However, a mitosporic fungus (Figure 6E–G) that resembled a reported anamorph of *Naemacyclus fimbriatus*, *Eriosporopsis albida* Petrak (Figure 6N,O) ([10]; [12] (pp. 77, 102); [54]), was found in a hand section made from the paratype. Unfortunately, the section was mounted in Melzer’s reagent and killed. Furthermore, before the mitosporic fungus was found, JMK had lightly crushed the section to try to spread the hymenial elements, which obliterated the structure of the section. JMK suspects that it was likely pycnidial and developed within the woody substratum under the apothecium. JMK was unable to find these again in the specimen and so was unable to attempt to establish a culture from these mitospores or sequence directly from sporomata. If these mitospores grew in culture, this would support considering them to be conidia. If they did not grow, one possibility is that the mitospores are spermatia.

Anamorphic states are reported for other taxa in Marthamycetales. In addition to *Naemacyclus fimbriatus*, these include *Cyclaneusma niveum* and *C. minus* [6], *Marthamyces harakeke* [20], and *M. johnstonii* [55]. These fungi produce more or less globose pycnidia that develop within plant tissues or on the surface of cultures, 150–600 µm diameter, with branching conidiophores supporting more or less cylindric phialides 5–17 × 1.5–3 µm, and produce filiform, straight–slightly curved mitospores 5–24 × 0.8–1.2 µm. Our observations of a possible mitosporic state of *R. octomerus* described above agree with these characteristics, although it does not seem that the phialides are produced on branching conidiophores but appear organized into a more or less palisade-like formation. We note that with the exception of Butin [6], none of the works treating the aforementioned fungi address the possibility that the mitospores could be spermatia; Butin uses the neutral term “pycnospores” but in the other contributions the mitospores are referred to as conidia. We will discuss this further in a subsequent contribution on the systematics of Marthamycetales.

### 5.4. Ecological Description of Collection Sites

The holotype of *Ramomarthamyces octomerus* (ILLS 00122394) was collected on La Gomera island, one of the smallest islands of the Canarian Archipelago (Macaronesia). It is situated in the Atlantic Ocean off the NW coast of Africa close to Morocco. The island is circular, with an area of 370.03 square kilometers, with rugged and steep coasts, particularly on the west, and a mountainous interior covered by a subtropical forest called laurel forest. Its climate is temperate and mild, with minimal fluctuation throughout the year, and with a rainy season during autumn-winter (Mediterranean climate). During the dry season, at altitudes between 600 to 1500 m, all islands experience a unique phenomenon known as horizontal rain, facilitated by the trade winds. These winds push moisture-laden clouds against the high mountainous terrain, causing the moisture to condense and precipitate horizontally. This provides an additional source of water during periods of low rainfall supporting the development of this forest at mid elevations in the archipelago. From the Tertiary period until the end of the Pliocene, in Europe and Southern Asia, there existed a forest with tropical affinities dominated by tree species similar to what we now call “laurisilva”, of which fossil remains still exist. During the Tertiary period, climate change and glaciations led to a progressive reduction of this vegetation, with the Iberian Peninsula being its last continental refuge. The laurel forest is now practically extinct in the continent but remains abundant in the Macaronesia region [56]. Today, one of the best-preserved laurel forests is found on La Gomera Island, inside the protected Garajonay National Park, which has been declared a World Heritage Site by UNESCO. This park occupies the central part of the island, which is covered by mature laurel forest and is rich in biodiversity, including numerous endemic species. Fungi play a key role in the laurel forest, and more than 50% of the macrofungi reported for Macaronesia inhabit this ecosystem (Beltrán-Tejera in [56]). Most fungal species found in the laurel forest are saprobes on woody substrates. Although there are several species found exclusively in the laurel forest (i.e., *Clitocybula wildpretii*, *Entoloma gomerense*, *Gymnopus beltraniae*, etc.), so far there are only a few examples from Leotiomycetes such as *Lambertella myricae* and *Moellerodiscus hederae* (op. cit.). *Ramomarthamyces octomerus* has so far only been found on La Gomera Island despite efforts to find it on other islands with Laurel forest. However, we consider that it is probably distributed in other islands with similar conditions to La Gomera Island, like La Palma and Tenerife. 

One paratype of *R. octomerus* (ILLS 00122395) was found in the Algarve region of southern Portugal. This region has a Mediterranean climate with typically dry summers and rainfall in the winter months (October to May, mostly in November and December). The collection site is situated in the hill land called “Barrocal“ where the soil is mainly composed of dolomitic limestone. The distance to the Mediterranean Sea (in the South) is about 20 km. The average yearly precipitation in the nearby town São Bartolomeu de Messines is not much more than 500 mm (climate-data.org). The twig covered with *R. octomerus* was collected in a dry microclimate (although located at the northern slope of a hill) with vegetation of many hairy and spiny Mediterranean flowers and shrubs growing under a very loose cover of trees and bushes such as *Quercus ilex* and cultivated trees such as *Olea europaea* (the plant associate), *Ceratonia siliqua,* and others. When the fungus was collected at the end of February 2022, it had not rained in the area for about 8 weeks after a period of strong rainfall in Dec 2021. The study area was completely dry and few other ephemeral fungi were collected.

These ecological conditions may be similar in the collection locality of specimen H.B. 6690 from the island of Korčula, Croatia. Although seated far more to the north than São Bartolomeu de Messines, the climate is also Mediterranean, and the soil is also composed of dolomitic limestone. Because Korčula is a small island, the distance from the collection locality to the Mediterranean Sea is short. Although the annual rainfall is quite high (about 1100 mm), most of the precipitation also occurs in the winter months (https://de.wikipedia.org/wiki/Korčula). This collection was made in June.

### 5.5. Morphology

#### 5.5.1. Amyloid Ascus Pores

The importance of determining the presence or absence of an ascus amyloid reaction in recognizing taxa in Ascomycota cannot be overstated. Indeed, early in our research for this contribution, the presence or absence of an ascus amyloid reaction in different specimens of *Ramomarthamyces octomerus* complicated recognition of these as belonging to the same species. Recently, notes and images of two geographically distant collections (holotype ILLS 00122394; paratype ILLS 00122395) were posted for discussion in the Ascomycota study forum AscoFrance (http://www.ascofrance.fr/search_forum/75419#). One of these collections had an amyloid apical ring (paratype ILLS 00122395, on *Olea europaea* from Algarve, Portugal) whereas the other did not (holotype ILLS 00122394, on *Laurus novocanariensis* from La Gomera). Baral [14] produced a provisional key, made available online, to the cylindric-ellipsoid-spored species of Marthamycetaceae that was widely circulated among members of the AscoFrance community, and this was used to key out these collections. Following the “Asci with amyloid apical ring” couplet, the specimen from Portugal keyed out to the provisional name “*Mellitiosporiella octomera*” (another provisional name for this fungus by HOB was “*Amylopropolis croatica*”) which was diagnosed based on a specimen also on *O. europaea* but from Dalmatia, Croatia (H.B. 6690, Figure 7(2)). Following the “Asci inamyloid” couplet for the specimen from La Gomera, this material keyed out to the provisional name “*M. canariensis*” that was diagnosed based on a specimen also on *L. novocanariensis* and from La Gomera (H.B. 6986, Figure 7(1)). In the AscoFrance discussion and in the notes accompanying his drawings, HOB considered that these two provisional species possibly represented the same fungus.

Prior to this contribution, an amyloid reaction in the asci of a Marthamycetales taxon had not been demonstrated. Baral et al. [1] characterized asci in Marthamycetaceae (and thus Marthamycetales because it is a monofamilial order) as “inamyloid, rarely with an amyloid ring”, with the as-yet unsequenced *Phragmiticola* being the exception. The other exception was the species described here as *R. octomerus*, which was placed in the family in the provisional key by Baral [14]. 

However, an amyloid ascus reaction had been previously reported in *Naemacyclus fimbriatus* that occurs on pine leaves and cones. Sherwood [7] (p. 42) examined a specimen of that species (on cones, Hungary, Zahlbruckner, CUP-D 107-47, as *Stictis fimbriata*) and stated that the ascus apices blued “faintly in iodine”. Sherwood [7] also cited Müller & Hütter [57] who likewise reported a bluing apical apparatus in a specimen on cones from Corsica. The microscopy methods used to arrive at this observation were not adequately described in these papers. Referring to the Materials and Methods section of Sherwood [30] (p. 6), specimens were typically rehydrated in “2% aqueous aerosol detergent solution” and Melzer’s reagent was preferred as a mounting medium. Potassium hydroxide (KOH) was also used to rehydrate some material, but this method was abandoned at some point in her research [30] (p. 6). We do not know if this approach was used in Sherwood [7]. We speculate that at some point after [7] was published, Sherwood became aware that asci of certain species directly mounted in iodine reagents might be inamyloid, but if these were rehydrated (pretreated) in KOH and then mounted in iodine reagents, an amyloid reaction would occur ([9] (p. 329); [58]). DiCosmo ([8]; [11] (p. 56)) did not observe this reaction in the specimens of *N. fimbriatus* that they studied on both leaves and cones from Europe and North America after rehydrating portions of hymenia in distilled water or 2% KOH and mounting these in Melzer’s reagent. HOB observed entirely thin-walled, inamyloid ascus apices using concentrated IKI (ca. 1% iodine) in recent collections of this species from France on both leaves (H.B. 7362) and cones (H.B. 4236, H.B. 6523), which is in keeping with the findings of DiCosmo et al. [11] (p. 56). Inamyloid asci (MLZ, KOH-pretreated) were also reported by Galán [59] for a Spanish collection on cones. JMK observed amyloid ascus pores in specimen ILLS 00122402 (on cones from Steuben, ME, USA) after pretreating material in 10% KOH followed by a rinse in tap water, then mounting in IKI (Figure 6M). JMK has not systematically tested the other specimens of *N. fimbriatus* examined in this contribution for amyloid ascus reactions with and without KOH pretreatment.

Baral [47] pointed out that amyloid reactions that were reported as requiring KOH pretreatment behaved differently between the two commonly-used iodine solutions: Melzer’s reagent and IKI. Ascus wall structures that required the pretreatment reacted negatively in just MLZ but red-brown in just IKI, but when pretreated with KOH gave a blue reaction in both reagents. This phenomenon, which Baral [47] called hemiamyloid, appears to occur also in *R. octomerus*. Hemiamyloidity was defined as encompassing not only the typical case described above but also the intermediate case in which ascus walls react blue at lower concentrations of iodine but dirty red at higher concentrations. When applying just MLZ to non-pretreated material, such walls react pale blue, but when pretreated they react strongly blue. We observed this intermediate type in H.B. 6690: the rings stained bright blue in IKI, but the reaction turned pale reddish gray at high concentration of the reagent.

Although we characterize *R. octomerus* as having amyloid ascus apical rings, we note that this character state is highly divergent between collections made on *Laurus* in the humid laurel forest of La Gomera, Canary Islands, and on *Olea* in the drier regions of southern Portugal and southern Croatia. Specimens from the humid locations have generally inamyloid ascus apices or only infrequently amyloid apical rings, and hymenial elements less agglutinated in gel, whereas those specimens from the latter locations had always strongly amyloid apical rings and hymenial elements firmly agglutinated in gel. We hypothesize that these morphological characteristics are related to differences in water availability between these localities.

The demonstration of an amyloid apical ring occurring in a taxon placed in Marthamycetales requires a revision of the morphological definition of this order. This will be addressed in a contribution to follow.

#### 5.5.2. Hymenial Gel

In addition to the differences in the iodine reactions of ascus rings between specimens collected in Portugal and Croatia, and those collected in the Canary Islands, Spain that are summarized in the Results section, we note that the paraphyses in the Portuguese and Croatian specimens were also firmly agglutinated in gel (Figure 5C) to the extent that the hymenial elements could not be readily spread in crush mounts, even after mounting in 10% KOH. This is in contrast to the specimens from the Canary Islands, where the hymenial elements were more or less loose, and though still somewhat difficult to spread in water, could be easily spread in 10% KOH. It was only in a senesced apothecium where we observed that the paraphyses were strongly agglutinated in gel.

We hypothesize that these differences in the agglutination of hymenial elements in gel are morphological adaptations to water availability. We are uncertain if apothecium age is a factor: both the holotype from La Gomera, Canary Islands and a paratype from southern Portugal readily discharged ascospores onto growth media for cultures and cover glasses for living ascospore observations (following the method given in Karakehian et al. [29]), although the two specimens differed in ascus amyloidity and the amount of hymenial gel. As discussed, the laurel forest ecosystem of the Canary Islands is a cloud forest that is very humid throughout most of the year due to rising winds from the sea, whereas the ecosystems of southern Portugal and Korčula island, Croatia are much drier throughout most of the year with monsoon rains occurring only during winter. Apothecia of *R. octomerus* are desiccation-tolerant and long-lived. When mature, they remain open to the elements even when dried out. That they fruit on dead exposed wood suggests that they are adapted to harsh environmental conditions including rapid drying and UV exposure [21].

#### 5.5.3. Paraphyses

Paraphyses in *Ramomarthamyces octomerus* are morphologically variable, even within a single apothecium. These may be more or less dichotomously branched near the top (Figure 5A,B). The apices may be simple or with a slight to moderate, capitate–clavate enlargement (Figure 7(1D,2D)). The apical branching is a synapomorphy among species of *Ramomarthamyces.* Johnston & Park [20] created the genus to accommodate a phylogenetic clade of *Marthamyces* species sharing this morphology.

#### 5.5.4. Ascospores

The ascospore morphology of *R. octomerus* is distinct from that of closely related species in Marthamycetaceae such as *Cyclaneusma*, *Marthamyces*, *Naemacyclus*, and *Ramomarthamyces*. Ascospores in species of these genera are filiform and equipped with gelatinous sheaths and caps at the poles, whereas the ascospores of *R. octomerus* are more or less cylindrical and lack gelatinous equipment, and the septa have small lateral thickenings. They are all similar in being smooth, hyaline, and possessing transverse septa. The results of our phylogenetic analyses are surprising because the ascospore shape of *R. octomerus* was suggestive of *Propolis*, and the septation pattern was strongly suggestive of *Mellitiosporiella*.

## 6. Conclusions

In this contribution, we describe *Ramomarthamyces octomerus* sp. nov. The fungus is described from collections made in Mediterranean climate zones in Europe and near northwestern Africa. Our phylogenetic placement of *R. octomerus* in Marthamycetaceae is based on maximum likelihood and Bayesian inference analyses of four genes. We characterize *R. octomerus* as having amyloid ascus apical rings but we note that this character state, and the degree to which the hymenial elements are agglutinated in gel, are highly divergent between collections made on *Laurus* in the humid laurel forest of La Gomera, Canary Islands, and on *Olea* in the drier regions of southern Portugal and southern Croatia. We hypothesize that these morphological characteristics are related to differences in water availability between these localities. Therefore, careful field notes on locality, collection date, and local climate are encouraged in future collections. We were unable to determine if a mitosporic fungus that we observed is the mitosporic state of *R. octomerus*, and if so, whether it is a conidial or spermatial morph. It is hoped that this contribution will facilitate future collections and study of this fungus that will increase our understanding of its distribution, morphological variation, ecological adaptation, host spectrum, and life history traits.

## Figures and Tables

**Table 1:** Isolates used in the phylogeny presented in this contribution. Number refers to that isolate in the phylogenetic tree given in Figure 2. GenBank numbers in bold type were newly generated for this study.

Number	Name	Herbarium No./Barcode	Culture	SSU	ITS	LSU	mtSSU	Plant Associate	Country
1	*Bulgaria inquinans*	NA	CBS 118.31	AJ224362	NA	DQ470960	NA	NA	NA
2	*Chaetomella oblonga*	BPI 843553	CBS 110.78	AY487084	AY487082	AY487083	NA	NA	NA
3	*Coccomyces dentatus*	OSC 100021	NA	AY544701	DQ491499	AY544657	AY544736	NA	NA
4	*Coccomyces proteae*	CBS H-20681	CBS 111703	NA	JN712451	JN712515	NA	NA	NA
5	*Cyclaneusma minus*	NA	CBS 496.73	FJ176812	NR_153910	FJ176868	FJ190629	Pinus radiata	Chile
6	*Cyclaneusma minus*	PDD 64257	ICMP 17358	KJ606669	KJ606680	KJ606674	MH698452	Pinus radiata	New Zealand
7	*Cyclaneusma niveum*	NA	CBS 495.73	**PP576077**	**PP576096**	**PP576086**	**PP554500**	Pinus nigra	Germany
8	*Cyclaneusma niveum*	ILLS 00122400	CBS 149209	**PP576070**	**PP576089**	**PP576079**	**PP554493**	Pinus sylvestris	Germany
9	*Leotia lubrica*	OSC 100001	NA	NG_013133	DQ491484	AY544644	AY544746	NA	NA
10	*Ramomarthamyces octomerus*	ILLS 00122394	CBS 150560	**PP576071**	**PP576090**	**PP576080**	**PP554494**	Laurus novocanariensis	Spain, Canary Islands
11	*Ramomarthamyces octomerus*	ILLS 00122395	CBS 150559	**PP576072**	**PP576091**	**PP576081**	**PP554495**	Olea europaea	Portugal
12	*Lophodermium pinastri*	NA	CBS 323.50	AF106014	NA	AY004334	AF431957	NA	NA
13	*Marthamyces* cf *emarginatus*	PDD 81847	ICMP 22621	MK681770	MK599212	MK599204	MK598752	Eucalyptus sp.	Australia
14	*Marthamyces culmigenus*	TNS F-41728	NA	NA	AB745435	AB745437	AB745436	Miscanthus sinensis	Japan
15	*Marthamyces desmoschoeni*	PDD 61761	ICMP 17350	KJ606670	KJ606679	KJ606673	OM201128	Desmoschoenus spiralis	New Zealand
16	*Marthamyces emarginatus*	PDD 81846	ICMP 22854	MK681769	MH921869	MK599203	MK598751	Eucalyptus sp.	Australia
17	*Marthamyces harakeke*	PDD 108765	NA	MK681771	MK599213	MK599205	MK598753	Phormium tenax	New Zealand
18	*Marthamyces maccormackii*	PDD 112256	ICMP 15829	OM201123	MK599217	OM201133	OM201130	Metrosideros collina	Cook Islands
19	*Marthamyces metrosideri*	PDD 82920	ICMP 17398	MH682226	MH682227	HM140558	HM143831.2	Metrosideros robusta	New Zealand
20	*Marthamyces quadrifidus*	PDD 43971	ICMP 18329	MK681772	MK599214	HM140559	HM143832	Weinmannia racemosa	New Zealand
21	*Marthamyces renga*	PDD 112257	ICMP 15830	OM201124	MK599221	OM201134	OM201127	Metrosideros collina	Cook Islands
22	*Microglossum rufum*	OSC 100641	NA	DQ471033	NA	DQ470981	NA	NA	NA
23	*Naemacyclus fimbriatus*	NA	CBS 289.61	**PP576078**	**PP576097**	**PP576087**	**PP554501**	NA	Switzerland
24	*Naemacyclus fimbriatus*	CBS H-20006	CBS 122316	NA	**PP576098**	**PP576088**	**PP554502**	Pinus pinea	Spain
25	*Naemacyclus fimbriatus*	ILLS 00122402	CBS 149212	**PP576073**	**PP576092**	**PP576082**	**PP554496**	Pinus banksiana	USA, Maine
26	*Naemacyclus fimbriatus*	ILLS 00122403	CBS 149213	**PP576074**	**PP576093**	**PP576083**	**PP554497**	Pinus rigida	USA, Rhode Island
27	*Naemacyclus fimbriatus*	ILLS 00122404	CBS 149214	**PP576075**	**PP576094**	**PP576084**	**PP554498**	Pinus banksiana	USA, Maine
28	*Phacidium pseudophacidioides*	NA	CBS 590.69	NA	KJ663853	KJ663894	NA	NA	NA
29	*Pilidium acerinum*	BPI 843555	CBS 736.68	AY487093	NA	AY487092	NA	NA	NA
30	*Potebniamyces pyri*	NA	NA	DQ470997	DQ491510	DQ470949	NA	NA	NA
31	*Propolis farinosa*	PDD 62678	ICMP 17354	MH682223	MH682229	HM140562	HM143834	Myrsine chathamica	New Zealand
32	*Propolis farinosa*	ILLS 00122401	NA	**PP576076**	**PP576095**	**PP576085**	**PP554499**	Fagus sylvatica	Austria
33	*Ramomarthamyces barbatus*	PDD 81891	ICMP 22853	MK681773	MH921868	MK599206	MK598749.2	Eucalyptus sp.	Australia
34	*Ramomarthamyces dracophylli*	PDD 44691	ICMP 17381	NA	MH682228	NA	MH698450	Dracophyllum sinclairii	New Zealand
35	*Ramomarthamyces gilvus*	PDD 81857	ICMP 22855	OM201125	MH921870	OM201132	OM201129	Caustis sp.	Australia
36	*Ramomarthamyces tuku*	PDD 62161	ICMP 22562	OM201126	MK599226	MK599208	MK598750	Juncus sp.	New Zealand
37	*Triblidiopsis pinastri*	NA	CBS 445.71	DQ471035	NA	DQ470983	AF431963	NA	NA
38	*Xeropilidium dennisii*	TU 104524	NA	KX090859	NA	KX090807	NA	NA	NA

**Table 2:** Comparison of selected features of *Ramomarthamyces octomerus* specimens obtained from six collection sites. * = living state, † = dead state, → apical thickening reduced from immature to mature. All values given in µm except for apothecium diameter that is given in mm. Double parentheses mean exceptional values for that collection. Footnotes: ^1^ Figure 5X, a weak amyloid reaction was observed by LQ in the isotype (TFC Mic. 25521), but JMK observed only negative reactions in all apothecia sampled from the holotype (ILLS 00122394). ^2^ JMK observed amyloid reactions in emptied asci in a senesced apothecium from the holotype (ILLS 00122394), although the apical rings could not be observed clearly because the asci were degraded (Figure 6L). ^3^ Reaction negative or weak when mounted directly in MLZ but stronger when MLZ added to water mount or when MLZ mount rinsed with water. ^4^ At higher iodine concentrations becoming dingy reddish. ^5^ Figure 5W.

	Spain, Canary Islands, La Gomera, Parque Nacional de Garajonay				Europe	
	H.B. 6986 (Chorros de Epina)	ILLS 00122394, TFC Mic. 25521, RN-14041504, RN-24012001 (Raso de la Bruma)	RN-23102901 (Los Baranquillos)	TFC Mic. 10068 (Agua de Los Llanos)	H.B. 6690 (Croatia, Korčula)	ILLS 00122395 (Portugal, Algarve)
Date	28 April 2001	18 March 2023, 15 April 2014, 20 January 2024	29 October 2023	28 April 2001	8 June 2000	22 February2023
Plant associate	*Laurus novocanariensis*	*Laurus novocanariensis*	*Laurus novocanariensis*	*Laurus novocanariensis*	*Olea europaea*	*Olea europaea*
Elevation	1000	1070–1090	1000	1000	45	200
Apothecium diameter	1.8–2.5 × 1.2–1.5	0.7–2 × 0.6–1.3			1.3–2.2 × 0.5–1	0.9–1.6 × 0.5–1
Ascospores	* 27–40 × 9–12.5 † 24–37 × 8.5–12	* (31.5–)37–43(–45) × 9.5–11.2 † 27.3–43.3 × 7.5–10.5		† 33–44.5 × 10–13	* 27–42 × 8.3–10.6 † (19–)23.5–36.5 × (7–)8–9.5	* 28.3–52 × 7.4–10.7 † 26.6–42.5 × 7.5–10.5
Ascospores, number of septa	7(–9)	(3–)7(–8)		(6–)7(–9)	(3–6–)7	3–7
Asci		† 125–177 × 17–25		† 136–177 × 17–23	† 93–140 × 12–21	† 127–167 × 15–21
Ascus apical wall thickness	† 0.2	† 0.4–0.5	† ~0.4–0.5		† 3.3 → 2–2.2	† 4–4.5 → 1.8–2.6
Ascus apex in MLZ	Inamyloid	Inamyloid			Amyloid (pale to strong bright blue ^3^)	Inamyloid or amyloid (light to bright blue ^3^)
Ascus apex in IKI	Inamyloid	Inamyloid	Inamyloid?	Inamyloid	Amyloid (strong blue and pale, dingy red ^4^)	Amyloid (strong dingy to bright blue ^5^)
Ascus apex in KOH pretreat + MLZ	Inamyloid	Inamyloid or weakly amyloid ^1^			Amyloid (strong, bright blue)	Amyloid (strong, bright blue)
Ascus apex in KOH pretreat + IKI	Inamyloid	Amyloid? ^2^	Inamyloid?	Inamyloid	Amyloid (strong, bright blue)	Amyloid (strong, bright blue)
Paraphyses apical cell	* 3.5–6 × 2.5–3.2	† 3.5–6 × 1.3–2.4			* 4–7.5 × 3.5–4.5	† 4–7.5 × 1.4–2
Periphysoids	* 25–40 × 3–4	* 25–33 × 2.5–3 † 22–36 × 1.8–3			* 22–25 × 2.5–3.5	† 17–24 × 2.1
Periphysoids, terminal cell	* 5–9 × 3–4	* 4.5–7 × 2.5–3			* 5.5–12 × 2.5–3.5	† 5.5–12 × 2.1
Stromatic tissue thickness		20–52				6–23

## Data Availability

Quantitative morphological data for the holotype and a paratype specimens of *R. octomerus* (ILLS00122394, ILLS00122395): https://datadryad.org/stash/dataset/doi:10.5061/dryad.83bk3jb0z.
